# Health in Germany: Establishment of a population-based health panel

**DOI:** 10.25646/11992.2

**Published:** 2024-03-13

**Authors:** Johannes Lemcke, Julika Loss, Jennifer Allen, Ilter Öztürk, Marcel Hintze, Stefan Damerow, Tim Kuttig, Matthias Wetzstein, Claudia Hövener, Ulfert Hapke, Thomas Ziese, Christa Scheidt-Nave, Patrick Schmich

**Affiliations:** Robert Koch Institute, Berlin, Department of Epidemiology and Health Monitoring

**Keywords:** PANEL, PUBLIC HEALTH SURVEILLANCE, HEALTH MONITORING, PROBABILITY SAMPLE, RECRUITMENT, PRIMARY DATA, DIGITISATION

## Abstract

**Background:**

The panel infrastructure Health in Germany, which is currently being set up, is geared towards the needs of public health research in Germany. The panel will consist of extensive probability and non-probability samples. This infrastructure will be used to collect survey data, measurement data and laboratory data to describe the health situation on an ongoing basis and make them available promptly.

**Methods:**

For the initial drawing of the probability sample, the sampling frame of the residents’ registration offices (EMA) established in Germany is used. The study design follows a mixed-mode approach in which the invited persons can choose whether to participate in the survey online or in paper form. Four surveys per year are planned for the regular operation of the panel (regular annual wave). Ad-hoc studies on specific topics or acute issues are also possible.

**Conclusions:**

The panel provides a new infrastructure for continuous epidemiological studies to monitor the health of the population in Germany. This data basis strengthens the health monitoring and health reporting of the federal government, enabling a prompt and adaptable response to emerging data needs.

## 1. Introduction and background

Reliable health-related data is required in order to develop needs-based and effective health and prevention policy measures. This makes it possible to describe the health situation of the population and identify health inequalities. Valid health information is therefore of great importance for the protection and promotion of health in all groups of the population. Federal health reporting is a central instrument for the provision of health-related information and is carried out by the Robert Koch Institute (RKI) together with the Federal Statistical Office. In addition to secondary data, such as official statistics and routine data from the social insurance institutions, the RKI’s own primary data from the RKI health monitoring system are also used as data sources. These include, for example, previous monitoring studies such as the German Health Interview and Examination Survey for Children and Adolescents (KiGGS) [1], the German Health Interview and Examination Survey for Adults (DEGS) [2] and German Health Update (GEDA) [3].

The COVID-19 pandemic has highlighted the need for continuous and timely data on the health of the population and specific population groups. The use of data was essential throughout the pandemic in order to classify the incidence of infection and its consequences and to derive targeted public health measures (e.g. for the public health service). In addition, various data collections were necessary to identify the effects on non-communicable diseases [4]. The pandemic has shown that health monitoring will have to change in the future: Times of crisis with acute threats to the health of the population require continuously up-to-date and promptly available population-based information in order to be able to quickly assess the health situation and act swiftly. At the same time, rapid feedback of the results to political decision-makers must be ensured. Even outside times of crisis, it is necessary to react quickly to new developments and issues in order to promote and protect the health of the population [4, 5]. There is also a great need for the development of target group-specific prevention measures and their evaluation.

Panel surveys are one way of collecting data quickly and flexibly in a randomly drawn sample (so-called probability sample) that can meet these requirements. In the case of a probability sample, a panel is a pre-recruited cohort of subjects who have agreed to participate regularly in studies on various topics [6]. Only the drawing of random, sufficiently large samples from an established sampling frame (which enables the calculation of inclusion probabilities) with a sufficiently large sample enables population-based (representative) statements to be made.

Over the next few years, a panel infrastructure consisting of extensive probability and non-probability samples will be established under the name of ‘Health in Germany’. Non-probability samples are based on self-selection by the participants and not on the random principle, e.g. target or occupational groups that are difficult to access. Using this infrastructure, three main types of data are to be collected on an ongoing basis and be available promptly: Survey data, measurement data (also referred to as examination data; information obtained through objective measurements by the subject or examiner, such as height and weight) and laboratory data (data based on extensive laboratory analyses which can be obtained, for example, through dried blood). The integration of these three types of data is realised through the use of new digital methods of data collection and data linkage. In further expansion stages, data linkage procedures (e.g. linking survey data and billing data from statutory health insurance companies) and data donation (e.g. wearable/fitness tracker data from participants that was not originally collected for research purposes but is made available to researchers through a voluntary ‘donation’ by the participants) are planned as central components. Data linkage and data donation studies are already established in research as pioneering extensions of epidemiological instruments [7, 8]. The participants (panelists) can also be asked about new or current health topics if required (so-called ad-hoc studies). In this regard, additional targeted data collection focusing on changes in health risks, behaviours and attitudes in the population is conceivable. Further data collection appears to be particularly useful in the context of rapidly changing living conditions or new findings, e.g. in connection with climate change and new findings on the causes of illness, prevention and treatment.

In principle, participants can be recruited to the panel in the course of sampling via two routes: firstly, via a random sample, i.e. via a probability sampling procedure, and secondly, via self-recruitment, i.e. via a non-probability sampling procedure (e.g. non-personalised calls on the internet [9]). Probability sampling methods are still the gold standard when it comes to generating generalisable population-based statements and estimates [10]. In the economic, social and behavioural sciences, probability panel infrastructures have become increasingly established in recent years. They are an integral part of the research infrastructure in both the commercial and public sectors. Probability participant cohorts and thus complex research infrastructures have already been successfully implemented in panel studies in other (social) scientific institutions, e.g. the GESIS panel (GESIS – Leibniz Institute for the Social Sciences) and the SOEP (Socio-Economic Panel at the German Institute for Economic Research, DIW) in Germany or the LISS panel (Longitudinal Internet studies for the Social Sciences) in the Netherlands [6, 11–14]. The second, non-probability recruitment route is particularly useful if subgroups in the population are to be included in a study that are not easily accessible via a conventional sampling framework (e.g. people with rare pre-existing conditions). In addition, this approach is useful for quickly recruiting control groups for case-control studies in small areas with an appropriate panel size [15]. Non-probability panels usually have a much larger number of participants, as potential participants can be approached more quickly through self-recruitment in conjunction with suitable advertising measures (e.g. via social media campaigns).

In addition to recurring cross-sectional surveys for population-based trend and time series analyses, a panel makes it possible to survey the same questions (e.g. health behaviour) at multiple points in time in the same sample. In this way, changes in individuals can be recorded over time, i.e. longitudinal analyses can be carried out. Population-wide longitudinal studies are of great importance for epidemiology and public health [16].

The underlying study design for the development of the Panel Health in Germany is presented below. In addition, the planned annual waves in the regular operation of the surveys in the panel and the expansion stages of the panel infrastructure for the coming years are described.

## 2. Study design of the panel recruitment

### 2.1 Sampling frame

In population-based epidemiological research in Germany, there are only a few practicable and efficient ways of drawing probability samples. These are, for example, sampling via existing official registers, such as the population registers of the residents’ registration offices (so-called EMA sampling frame), or sampling via a generated telephone sampling frame (RDD, Random Digit Dialing). Each of these sampling frames has advantages and disadvantages (see [17], among others). For the telephone sampling frame and the associated telephone survey mode, as for face-to-face surveys, the so-called response rate is comparatively low and has been stagnating or declining for years [18, 19]. The risk of a non-response bias is therefore often higher. In contrast, higher recruitment rates can be achieved when using official registers, such as the residents’ registration offices (EMA), and the non-response bias, particularly in form of an educational bias, is less pronounced [20]. In addition, the use of the EMA sampling framework accommodates the design of a self-administered panel, in which respondents take part in the surveys online or via paper questionnaires. This means that participants do not have to switch from a telephone mode of recruitment to a self-administered mode, which reduces the risk of substantial selection effects. The disadvantage of recruitment via residents’ registration offices is that the selection is made via sample points, which can result in a cluster effect. However, this is less significant in the overall view. Due to the expected higher probability of participation, it was decided to carry out the initial recruitment for the Panel Health in Germany using the EMA sampling framework as a basis.

### 2.2 Sample

The target (reference) population for the panel recruitment is people aged 16 and over usually residing in private households in the Federal Republic of Germany during the survey period. The sample was drawn in cooperation with GESIS in Mannheim. A two-stage, stratified (cluster) sample was drawn and the persons drawn were invited to participate. For this purpose, 359 Primary Sampling Units (PSUs), so-called sample points, were randomly drawn from the total number of all political municipalities in Germany (first selection stage). The drawing was proportional to the number of inhabitants and stratified according to federal state and BIK municipality size class (a regional classification system for Germany [21]), so that the regional structure of Germany is adequately represented. In the second selection stage, addresses were drawn for each sample point stratified by age group from the address registers of the respective residents’ registration offices using a statistical random procedure (unrestricted random selection).

The aim is to recruit a minimum number of 30,000 active panelists. The basis for this is the requirement that nationwide estimates for health parameters (e.g. proportion of the population in Germany with a chronic illness), stratified by gender and age group, can be estimated with sufficient statistical accuracy by the time the refreshment sample is drawn after two years. The following assumptions were made for the case number estimates: The nominal estimated number of cases after two years results from a panel attrition (withdrawal of panel participants) of 25% and a participation rate of 65% of active panelists [22]. In addition, design effects due to the increase in small federal states, the clustered study design [23], as well as drop-out weighting and adjustment weighting to public statistics were considered. The design effects are derived from experience with previous RKI surveys. The anticipated total design effect (product of all design effects) is estimated to be approximately two, depending on the number of sex and age groups. The effective number of cases, which determines the statistical accuracy, results from the nominal estimated number of cases divided by the total design effect. Based on these assumptions, a panel size of 30,000 active participants was calculated as the optimal size, as the resulting effective number of cases can be used to estimate, for example, stratum-specific prevalences with two gender and four age groups of 5% with sufficient accuracy (95% Wilson confidence interval 3.8%–6.6%; details in Annex Table 1).

The gross sample (i.e. the complete list of all addresses drawn in this way) is divided into three portions and sequentially incorporated into the recruitment process. In an initial run-in phase, which is intended to evaluate and test the processes, 10% of the gross sample is contacted (i.e. approx. 10% of the addresses). Following that, two main segments (segment 1 comprising 50% and segment 2 comprising 40% of the addresses) are sequentially processed.

### 2.3 Survey design of the recruitment study

Recruitment is based on a so-called mixed-mode approach [13, 24–26]. The invited persons can choose which survey mode they use to participate in the initial panel survey, the so-called welcome survey with the subsequent option of panel registration.

**Figure 1:**
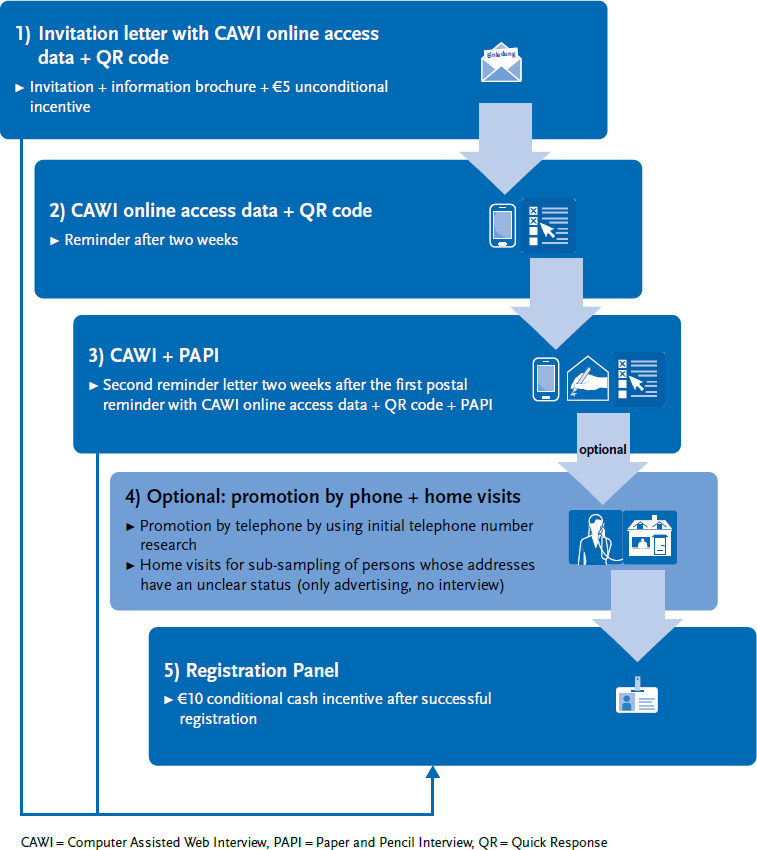
Overview of the invitation/recruitment process for the first recruitment study Source: Own figure

Figure 1 shows the basic structure of the procedure in simplified form.

The sequence of the survey modes offered is differentiated on the basis of the age groups (which are available via the EMA framework information for selecting the sample). The following differentiation is planned:

‣ Age group 16–69 years: Sequential mixed-mode design (push-to-web strategy) in the order shown in Figure 1. Here, the people invited are first given the opportunity to take part in the survey online (CAWI – Computer Assisted Web Interview). Only with the second reminder is a paper questionnaire (PAPI – Paper and Pencil Interview) offered.
‣ Age group 70+ years: Simultaneous mixed-mode design; CAWI and PAPI are offered from the outset.

#### 2.3.1 Invitation procedures

##### Invitation letter

As initial contact, all persons in the gross sample receive a postal invitation letter. This contains a cover letter, an information brochure and an unconditional €5 cash incentive. As already mentioned, the letter for the age group 16 to 69 years initially only contains the online access information for the CAWI mode. In the 70+ age group, a simultaneous offer is made directly, which contains the online access details for the CAWI mode and a paper questionnaire for the PAPI mode. The reason for this is that the average internet usage rate in the older age group is still lower than in younger age groups [27]. Without a simultaneous offer, the effectiveness of the unconditional incentive could be reduced due to the lower internet usage. This is because the age groups in question could not immediately fulfill the norm of ‘social exchange’ in this case (more details on the ‘social exchange’ norm are explained in section 2.3.4 ‘Incentive concept’).

##### First postal reminder

The first postal reminder is sent out after two weeks. There is no differentiation between the age groups here. The reminder letter again contains the CAWI access information as a link and QR code, but no paper questionnaire.

##### Second postal reminder

The second postal reminder is sent two weeks after the first postal reminder with the CAWI access information as a link and QR code. The paper questionnaire, i.e. the PAPI mode, is now also offered to the 16 to 69 age group. In the 70+ age group, the paper questionnaire will be sent out again.

#### 2.3.2 Promotion phase (optional)

In this phase, it is possible to carry out further optional promotional measures. These can primarily serve to improve the sample composition. Improving the sample composition here means above all minimising a possible non-response bias (i.e. a large difference between subgroups in the sample and in the true population distribution). This bias has been documented, especially for educational groups [28]. Furthermore, they can help to clarify the status of people in the gross sample who have not yet been reached.

##### Promotion by telephone

Promotion by telephone is used after the two reminder letters for the remaining unresolved cases, i.e. for addresses without information on status. This measure is based on an initial telephone number search directly after the EMA sample has been drawn. For this purpose, a service provider will match address data and landline numbers via databases. Experience has shown that a telephone number can only be researched for around 20% to 25% of addresses. The proportion is significantly higher in the 65+ age groups. The aim of this measure is not primarily to increase the response rate, but rather to classify the previously unsolved cases in order to reduce the workload for the subsequent home visit by reducing the number of addresses to be contacted. If the person selected during the sampling process is successfully contacted during telephone canvassing, participation in the study is advertised. A telephone interview is not planned.

##### Home visit

This measure is also optionally implemented depending on the effectiveness of the previously implemented measures. Here, home visits are to be made to a sub-sample of those people whose addresses still have an unclear status up to this phase in order to advertise participation in the study. Conceptually, it is planned that contact via home visits will be limited to hard-to-reach groups [29, 30], i.e. groups that are difficult to reach for studies (e.g. older people aged 80 and over, younger and therefore often mobile people). These groups are not determined a priori before the start of the fieldwork, but are defined if necessary after considering initial results on the sample composition from the run-in phase.

#### 2.3.3 Panel registration

##### Welcome survey and registration

Panel registration takes place after the welcome survey. This survey should give respondents a first impression of the content of the panel, be entertaining and have a motivating effect on their willingness to participate in surveys again. The maximum targeted completion time should not exceed ten minutes. This welcome survey can also be used to answer content-related questions with a large number of cases (estimated sample size over 50,000). The number of participants in the welcome survey will be larger than the number of people who subsequently register for the panel, as it can be assumed that not all participants in this survey will be willing to be interviewed again. In addition, by collecting initial data on the health status, health behaviour and basic sociodemographic of the respondents, selection effects during the panel registration can be analysed. In this way, a drop-out weighting for the subsequent survey waves can be carried out on the basis of model estimates.

After completing the welcome survey, online participants who are willing to be interviewed again are redirected to the registration page of the panel portal. Here they enter their name, e-mail address, postal address, optional telephone number, date of birth and gender and create an account. They then receive an automated verification e-mail. This e-mail contains a confirmation link that leads to the final registration in the panel (double opt-in procedure). From this point on, the participants registered online are considered active panelists who are available for regular panel operation.

Participants who register for the panel via the PAPI mode go through a slightly modified registration process. The relevant registration parameters (name, gender, date of birth, postal address, optional telephone number) are imported into the panel management software on the basis of the returned declaration of consent for panel participation and at this point the panelists participating offline are marked as ‘offline’ so that re-contacting can take place directly with a paper questionnaire during regular panel operation. It should be possible to switch from participation with a paper questionnaire to participation via an online survey.

#### 2.3.4 Incentive concept

The use of incentives as an extrinsic incentive to encourage study participation has been extensively studied within survey research. A large number of studies and literature reviews have shown that the use of incentives has a positive effect on survey response rates. A comprehensive meta-analysis of 251 postal surveys has shown that offering a cash incentive doubles the probability of participation [31]. In addition, this positive effect can be transferred to other survey modes [32]. Incentives have also been successfully used in recruitment for panel infrastructures [11, 33, 34].

It also shows that there is a difference in the efficiency of different types of incentives on the response rate. For example, monetary incentives are more effective than non-monetary incentives, such as participation in a lottery for participating in the study [35]. In addition, unconditional incentives are often much more effective in increasing response rates than conditional or contingent incentives [36]. A positive non-linear relationship between incentive level and response rate has also been observed in many cases [37]. These empirical results can be explained by the theory of social exchange [38]. According to this theory, social exchange is based on reciprocity. A ‘gift’ can create an obligation to reciprocate. In survey studies, an unconditional incentive prior to the actual participation in the study can act as such a ‘gift’, making the reciprocation through study participation more likely.

Based on this research situation, the following incentive concept was developed for the initial recruitment of the panel: In the invitation procedure described above, an unconditional cash incentive of €5 is used. This is sent in the invitation letter at the initial contact. Furthermore, after registration (for online participants) or after returning the consent form (for offline participants), a conditional incentive of €10 is sent to respondents.

## 3. Regular panel operation

**Figure 2:**
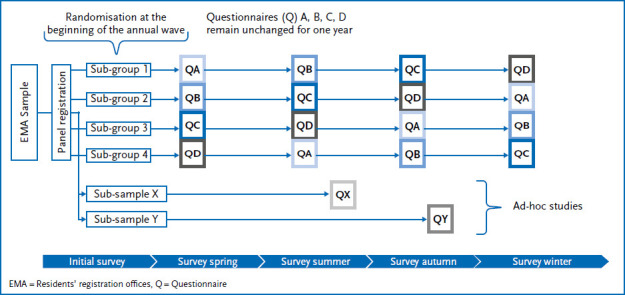
Illustration of the panel structure in regular operation Source: Own figure

The first recruited cohort from the German-speaking population of at least 30,000 participants will be available for regular operation in spring 2024. This will consist of the regular annual wave and additional ad-hoc studies (Figure 2):

‣ The annual wave consists of four sub-waves per year and surveys key indicators of health monitoring, i.e. indicators from the areas of physical and mental health, health and utilisation behaviour as well as social determinants of health. The survey is spread evenly over four survey periods covering the four seasons in order to be able to control for seasonal effects on prevalence estimates. This is made possible by a randomised split of the entire panel sample into four sub-groups at the beginning of a survey period. The groups each receive four different questionnaires in a rotating order at four different points in time. This prevents all participants from answering certain questions in only one quarter, which is particularly relevant for indicators with seasonal fluctuations (e.g. physical activity, sleep, mental health). By splitting up, estimates for such indicators are obtained from different seasons and cumulated at the end of a survey period (here: one year). However, this rotation procedure carries the risk of increasing the probability of incomplete data sets due to inconsistent participation patterns.
‣ In ad-hoc studies, panel participants are invited to take part in further in-depth studies flexibly and at short notice. These studies should include current public health issues, e.g. in relation to a new health policy intervention or a new social development that may act as a stressor or, for example, on questions and attitudes regarding the use of early detection. The scope of ad-hoc studies that can be carried out each year depends on the current workload of the panel. If necessary, these studies can also be linked intra-individually with the content of the annual waves and thus offer the opportunity to obtain a more comprehensive picture of the health situation of the surveyed participants. There is currently no precise empirical evidence on the optimal number of studies per year that should be offered for each participant in a panel infrastructure. Although there are recommendations and strategies, such as those of the AmeriSpeak Panel of the University of Chicago (NORC), which plan with one study invitation per week [39], these are not based on experimental research. In general, it is important to ensure that the respondents are neither overburdened nor ‘underchallenged’. Both can have a negative effect on the motivation to participate and thus on the probability of participation.

### 3.1 Panel maintenance

Panel management is constantly faced with the challenge of taking suitable measures to maintain the panel. Panel infrastructures face the particular challenge of retaining the participants recruited in the long term [40]. This is the only way to ensure longitudinal studies of individuals over time and thus amortise the initial recruitment costs in further follow-up studies.

Measures are planned to counteract panel attrition, i.e. the dropping out of participants, and panel conditioning, i.e. the learning effect of changing response behaviour due to repeated participation:

#### 3.1.1 Refreshment samples

In panel surveys, refreshment samples are often used to increase the sample size overall or for certain groups and to improve representativeness [41]. In the first wave of data collection, panel surveys provide the same information as one-off cross-sectional surveys, and from the second wave onwards they also provide cross-sectional variations. However, they may not measure the current population, as the composition of the population is potentially no longer the same as at the time of the first sampling. For these reasons, the integration of a refreshment sample as a new cohort into the existing panel is planned every two years. As the composition of the population can change even in relatively short periods of time, current developments, e.g. through immigration and emigration, births and deaths, should also be reflected in the panel. This refreshment sample follows a similar procedure and system to the initial recruitment process described above.

#### 3.1.2 Incentive programs to increase the probability of participation

##### Incentives per study

Incentives are also considered a suitable instrument for continuous panel maintenance in order to increase the probability of re-participation [42, 43]. The special feature is that the positive effect on the willingness to participate can remain effective over several follow-up waves, thus keeping panel attrition low [44]. For the follow-up waves, panel participants receive incentives to be determined more precisely for each invitation and for each successful participation in the surveys of the annual waves. For participation in ad-hoc studies, these incentives may differ and will primarily depend on the available budget and the planned number of cases.

##### Panelist support – community management and tracing measures

Active community management is an important task for panel maintenance. The guiding principle is to offer participants an appreciative, motivating and enriching experience [45]. Community management is to be controlled via various channels. For example, a telephone hotline will be set up, the capacity of which will be increased during active studies, especially during regular annual waves. The hotline covers both technical and content-related questions. In addition, e-mail support will be set up for written digital inquiries. A catalog of frequently asked questions (FAQs) will be compiled gradually in order to answer participants’ queries and will be continuously supplemented with further information. An FAQ section will also be included on the website https://www.gesundheit-in-deutschland.de, which contains information for participants and the access link to the survey platform. This information will be continuously updated and extended. Regular communication on the content of the panel surveys will take place via social media channels, e.g. X (formerly Twitter) and Instagram. The following formats have proven successful in communicating with study participants: Sending a newsletter with current topics, sending personalised birthday wishes and Christmas greetings, regular invitation to update profile data and request for confirmation of current postal address as well as communication of a ‘number of the month’ from results of current studies and ‘statistics of the week’ as a weekly format. The last two measures are also used in press and public relations work. In addition, the regular updating of addresses via queries to the residents’ registration offices plays an important role.

## 4. Contents of the panel surveys

### 4.1 The Panel Health in Germany as the cornerstone for health monitoring

The data collection in the Panel Health in Germany will make a key contribution to the expansion of indicator-based health monitoring by the federal government by continuously gathering epidemiological data on the health status of the population that is not otherwise available. The selection of topics relevant to health policy includes indicators of physical and mental health, health behaviour, the use of health services and social determinants of health and follows a framework concept similar to the European health indicators (ECHI) [46]. For some topics, e.g. diabetes mellitus [47, 48], mental health [49] and childhood obesity [50], a limited number of indicators were selected in a structured expert consensus. The selection of a set of core indicators lays a further foundation for the development of public health surveillance in Germany. In recent years, the RKI has not only greatly expanded its surveillance activities in the area of infectious diseases, but has also created a basis for the development of surveillance activities for non-communicable diseases in cooperation with international partners, e.g. in the context of diabetes and mental health surveillance [49, 51, 52]. These activities are to be gradually extended to the systematic surveillance of other diseases (e.g. cardiovascular diseases) and important common risk factors. Consensual core indicators on the state of health of the population and on individual and social determinants of health are to be collected at regular intervals from 2025 in the Panel Health in Germany, with the survey frequency varying for individual indicators. Together with information from relevant and regularly available secondary data, they are intended to provide an overview of the most important key data on population health.

### 4.2 Survey topics in 2024

The first annual wave of the Panel Health in Germany in 2024 will contain key data for health reporting. Central topics are information on self-assessed health status, the prevalence of chronic illnesses, restrictions in everyday life and physical functioning. These core indicators are to be collected annually or every two years. In order to monitor the development of mental health in the population, key symptoms of mental disorders (depression, anxiety disorders and post-traumatic stress disorder) and aspects of positive mental health (well-being, quality of life) are surveyed. The panel surveys also include factors that have a decisive influence on mental health, e.g. chronic stress and resources such as resilience. In addition, various subjectively perceived stressors, e.g. concerns about the effects of climate change, environmental protection or job security, are surveyed. Another block of questions deals with healthcare, in particular with the question of whether the participants feel well cared for or whether there are unmet health care needs. The aim is to investigate which barriers make it difficult to make use of the services, e.g. in the case of mental health problems. For the first time in over ten years, accidents and their personal and social consequences will also be recorded. In connection with the so-called post-COVID syndrome, population-based information on post-pandemic symptom burdens is also required. Questions are therefore planned, for example, on restrictions in cognitive function, sleep quality and fatigue. The focus topics in 2024 are the general health literacy and nutritional literacy of the population, which are of current relevance against the backdrop of the German government’s currently planned National Prevention Plan on the one hand and the planned nutrition strategy on the other.

Physical and mental health must always be seen in the context of influences such as social support and social isolation (loneliness), which will be surveyed in the panel in 2024. Family situation, educational status, income and migration status are also surveyed. Health-threatening stresses associated with current financial difficulties and conditions in the world of work are also recorded. This makes it possible to identify the social groups in which there is a particular need for prevention and support.

### 4.3 Prompt communication of the results to policy-makers, practitioners and scientists

The planned data collection, which will be carried out as part of the panel infrastructure, can be used to develop and evaluate prevention measures at population level. In-depth topics of current interest can be introduced as part of the ad-hoc studies. The COVID-19 pandemic has made it clear that rapid feedback from epidemiological analyses to political decision-makers is important. The results should be made available to stakeholders in politics, research and healthcare in a timely manner in order to identify the need for action and research and to support the implementation and impact of public health measures. To this end, the Health Information System (HIS) is currently being developed as a new format for presenting and communicating findings from health monitoring. The core of the HIS is an online platform that visualises the results in a structured, clear and descriptive manner and explains them in a way that is appropriate for the target audience.

## 5. Planned expansion stages

**Figure 3:**
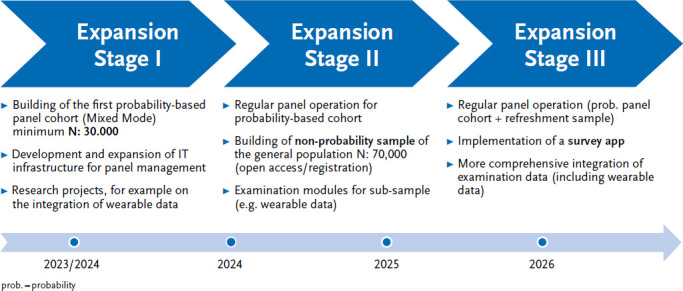
Expansion stages of the Panel Health in Germany Source: Own figure

For the panel infrastructure described in this article, an expansion plan was developed for the coming years, which provides for extensions to data collection, access to participants and the addition of further data sources (e.g. routine data) (Figure 3). Following the establishment of regular operation of the annual waves for the survey content of the health monitoring (expansion stage I), examinations and measurements (e.g. blood sampling, blood pressure measurement, anthropometry) are also to be carried out on a sub-sample in the future (expansion stage II). A concept for the implementation of examination modules is currently being developed. In this expansion stage, it is also planned to open up the panel for self-recruitment of a non-probability sample. The central element in the third expansion stage is the development of a central survey app for smartphone users. Figure 3 shows the most important milestones to be achieved over the next few years as a rough guide. Not mentioned here are further expansion stages, such as the integration of a sample of children and adolescents.

## 6. Conclusion and prospects

The establishment of the Panel Health in Germany marks a significant advance in epidemiological research and fulfills the increased requirements for public health surveillance for non-communicable and communicable diseases. This panel represents a new instrument for the public health landscape in Germany, which not only offers a comprehensive sample, but also allows flexible expansion to include new topics. A specific advantage of this panel is its scalability in principle. This opens up the possibility of integrating research questions and study content from other scientific institutions. A key feature of the panel is the wealth of data on various topics, including social, health and behavioural aspects. These data are not isolated, but can be linked together, which opens up the possibility for more in-depth analyses. The potential for longitudinal studies significantly expands the range of possible research questions and allows changes over time to be analysed. In addition, specific subgroups can be selected for studies using the available profile data on the panel participants. Health in Germany also offers great potential for health reporting and surveillance: the flexibility of the panel will enable health reporting to provide more up-to-date and differentiated analyses of important public health trends and new public health-relevant issues in the future. To this end, suitable digital information services such as dashboards or interactive evaluation tools for key public health indicators are to be developed and made available.

Despite the great potential for public health research, there are some challenges that need to be considered: Organisationally, panel maintenance requires continuous management and a stable resource base to ensure that the panel remains effective and representative over time. In this context, selective panel attrition is one of the biggest challenges. In addition, there are possible sources of error due to repeated interviews with the same participants (so-called panel conditioning). These aspects must be continuously monitored. Ways of meeting these challenges have been identified in this text.

The Panel Health in Germany has the potential to be the central instrument for the public health research landscape with regards to monitoring population health in Germany. It not only serves to protect and promote the health of the population, but can also be adapted quickly and flexibly to acute needs in crisis situations, e.g. through high-frequency or additional data collection to answer urgent questions. The panel can also serve as a basis for digital data collection, e.g. by linking it to wearables.

The task of operating the ‘Health in Germany’ study series is expected to be transferred to the Federal Institute for Prevention and Education in Medicine (BIPAM) in 2025. As part of a reorganization of the authorities in the subordinate area of the Federal Ministry of Health, the focus of the newly established BIPAM will be on the prevention of non-communicable diseases, while the Robert Koch Institute will focus on infectious diseases.

**Corrigendum, page 16**

This article was initially published without an acknowledgement, this has now been added by the authors.

## Key statement

- Health policy and research must recognise changes and the need for action at an early stage. This requires population-based health data that can be continuously compared over time.
- In addition to trend and time series analyses, panel surveys financed over the long term also enable longitudinal individual progression analyses.
- Long-term financed panel infrastructures enable and facilitate the use of innovative forms of data collection.
- The regular panel operation consists of the regularly conducted annual wave and additional ad-hoc studies.
- Based on the current research situation, a comprehensive incentive concept was developed for the first panel recruitment.

## Figures and Tables

**Annex Table 1:** Case number calculation Source: Own table

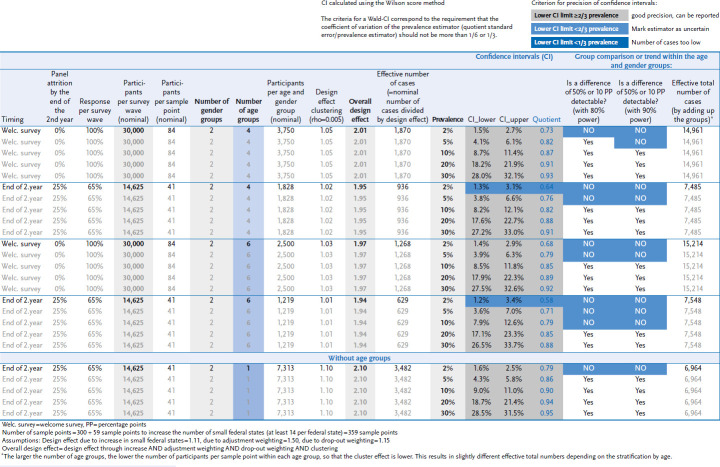

## References

[1] Mauz E Lange M Houben R(2019) Cohort profile: KiGGS cohort longitudinal study on the health of children, adolescents and young adults in Germany. International Journal of Epidemiology 49(2):375–375k 10.1093/ije/dyz231 PMC7266535 31794018

[2] Gößwald A Lange M Dölle R(2013) Die erste Welle der Studie zur Gesundheit Erwachsener in Deutschland (DEGS 1). Robert Koch-Institut, Epidemiologie und Gesundheitsberichterstattung. Bundesgesundheitsbl 56:611–619 10.1007/s00103-013-1671-z 23703477

[3] Allen J Born S Damerow S(2021) German Health Update (GEDA 2019/2020-EHIS) – Background and methodology. J Health Monit 6(3): 66–79. https://edoc.rki.de/handle/176904/8757 (As at 19.02.2024) 35146317 10.25646/8559 PMC8734110

[4] Shadbolt N Brett A Chen M(2022) The challenges of data in future pandemics. Epidemics 40:100612 35930904 10.1016/j.epidem.2022.100612 PMC9297658

[5] Sachs JD Karim SSA Aknin L(2022) The Commission on lessons for the future from the COVID-19 pandemic. The Lancet 400(10359):1224–1280 10.1016/S0140-6736(22)01585-9 PMC9539542 36115368

[6] Bosnjak M Dannwolf T Enderle T(2017) Establishing an Open Probability-Based Mixed-Mode Panel of the General Population in Germany. Social Science Computer Review 36:103–115

[7] Chaturvedi RR Angrisani M Troxel WM(2023) American Life in Realtime: a benchmark registry of health data for equitable precision health. Nat Med 29(2):283–286 36737671 10.1038/s41591-022-02171-w PMC9974879

[8] Radin JM Wineinger NE Topol EJ(2020) Harnessing wearable device data to improve state-level real-time surveillance of influenza-like illness in the USA: a population-based study. Lancet Digit Health 2(2):E85–E93 33334565 10.1016/S2589-7500(19)30222-5 PMC8048388

[9] Martin-Luther-Universität Halle-Wittenberg (2023) DigiHero Hintergründe. https://webszh.uk-halle.de/digihero/hintergruende/ (As at 24.10.2023)

[10] MacInnis B Krosnick JA Ho AS(2018) The Accuracy of Measurements with Probability and Nonprobability Survey Samples: Replication and Extension. Public Opinion Quarterly 82(4):707–744

[11] Blom AG Bosnjak M Cornilleau A(2015) A Comparison of Four Probability-Based Online and Mixed-Mode Panels in Europe. Social Science Computer Review 34:8–25

[12] Cornesse C Felderer B Fikel M(2021) Recruiting a Probability-Based Online Panel via Postal Mail: Experimental Evidence. Social Science Computer Review 40:1259–1284

[13] Dollmann J Mayer SJ Lietz A(2022) DeZIM.panel – Data for Germany’s Post-Migrant Society. Jahrbücher für Nationalökonomie und Statistik 243:93–108

[14] Wagner GG Göbel J Krause P(2008) Das Sozio-oekonomische Panel (SOEP): Multidisziplinäres Haushaltspanel und Kohortenstudie fürDeutschland – Eine Einführung (für neue Datennutzer) mit einem Ausblick (für erfahrene Anwender). AStA Wirtschafts- und Sozialstatistisches Archiv 2(4):301–328

[15] Mook P McCormick J Kanagarajah S(2018) Online market research panel members as controls in case-control studies to investigate gastrointestinal disease outbreaks: early experiences and lessons learnt from the UK. Epidemiol Infect 146(4):458–464 29332618 10.1017/S0950268817002953 PMC5848756

[16] Deutsche Akademie der Naturforscher Leopoldina e. V. (Ed) (2016) Wissenschaftliche und gesellschaftspolitische Bedeutung bevölkerungsweiter Längsschnittstudien. Schriftenreihe zur wissenschaftsbasierten Politikberatung: Stellungnahme. Druckhaus Köthen GmbH & Co. KG

[17] Häder S (2015) Stichproben in der Praxis (Version 1.1). GESIS Survey Guidelines:17

[18] Dutwin D Lavrakas P (2016) Trends in Telephone Outcomes, 2008–2015. Survey Practice 9(3)

[19] Leeper TJ (2019) Where Have the Respondents Gone? Perhaps We Ate Them All. Public Opinion Quarterly 83(S1):280–288

[20] Lange C Finger JD Allen J(2017) Implementation of the European health interview survey (EHIS) into the German health update (GEDA). Arch Public Health 75:40 28936356 10.1186/s13690-017-0208-6 PMC5603169

[21] Arbeitsgruppe Regionale Standards (Hrsg) Regionale Standards. Ausgabe 2019. 3. überarbeitete und erweiterte Auflage. GESIS-Schriftenreihe, Vol 23. https://www.destatis.de/DE/Methoden/Demografische-Regionale-Standards/Downloads/regionale-standards-2019.pdf?__blob=publicationFile (As at: 06.03.2024)

[22] Kaczmirek L Phillips B Pennay D(2019) Building a probability-based online panel: Life in Australia. Methods Paper No. 2/2019 ANU Centre for Social Research & Methods Research School of Social Sciences. The Australian National University

[23] Wiegand H (1968) Kish, L.: Survey Sampling. John Wiley & Sons, Inc., New York, London. Biometrische Zeitschrift 10(1):88–89

[24] Bujard M Gummer T Hank K(2022) FReDA – Das familiendemographische Panel FReDA – Das familiendemographische Panel. GESIS

[25] Dillman DA Smyth JD Christian LM (2014) Internet, phone, mail, and mixed-mode surveys: The tailored design method. John Wiley & Sons, Hoboken, New Jersey

[26] Horwitz R Lesser V Marken S(2019) Report of the AAPOR Task Force on Transitions from Telephone Surveys to Self-Administered and Mixed-Mode Surveys. https://aapor.org/wp-content/uploads/2022/11/Report-of-the-Task-Force-on-Transitions-from-Telephone-Surveys-FULL-REPORT-FINAL.pdf (As at 11.03.2024)

[27] Destatis (2023) Internetnutzung für private Zwecke. https://www.destatis.de/DE/Themen/Gesellschaft-Umwelt/Einkommen-Konsum-Lebensbedingungen/IT-Nutzung/_inhalt.html#sprg229130 (As at 19.02.2024)

[28] Mindell JS Giampaoli S Goesswald A(2015) Sample selection, recruitment and participation rates in health examination surveys in Europe – experience from seven national surveys. BMC Med Res Methodol 15:78 26438235 10.1186/s12874-015-0072-4 PMC4595185

[29] Bacher J Lemcke J Quatember A(2019) Probability and nonprobability sampling: Representative surveys of hardto-reach and hard-to-ask populations. Current surveys between the poles of theory and practice. Survey Methods: Insights from the Field. https://surveyinsights.org/?p=12070 (As at 11.03.2024)

[30] Tourangeau R (Ed) (2014) Hard-to-survey populations. Cambridge University Press

[31] Edwards P Roberts I Clarke M(2002) Increasing response rates to postal questionnaires: systematic review. BMJ 324(7347):1183 12016181 10.1136/bmj.324.7347.1183 PMC111107

[32] Medway RL Fulton J (2012) When More Gets You Less: A Meta-Analysis of the Effect of Concurrent Web Options on Mail Survey Response Rates. Public Opinion Quarterly 76(4):733–746

[33] Blom AG Gathmann C Krieger U (2015) Setting Up an Online Panel Representative of the General Population. Field Methods 27(4):391–408

[34] Struminskaya B (2014) Data quality in probability-based online panels: Nonresponse, attrition, and panel conditioning. Utrecht University Repository

[35] Petrolia DR Bhattacharjee S (2009) Revisiting Incentive Effects: Evidence from a Random-Sample Mail Survey on Consumer Preferences for Fuel Ethanol. The Public Opinion Quarterly 73(3):537–550

[36] Singer E Ye C (2013) The Use and Effects of Incentives in Surveys. The ANNALS of the American Academy of Political and Social Science 645(1):112–141

[37] Mercer A Caporaso A Cantor D(2015) How Much Gets You How Much? Monetary Incentives and Response Rates in Household Surveys. Public Opinion Quarterly 79(1):105–129

[38] Gouldner AW (1960) The Norm of Reciprocity: A Preliminary Statement. American Sociological Review 25(2):161–178

[39] Dutwin D (2023) When Does Use Become Abuse in Panels? Considering Burden. NORC Research Brief. https://www.norc.org/content/dam/norc-org/pdfs/Panelist%20Burden%20Research%20Brief.pdf (As at 11.03.2024)

[40] Lugtig P (2014) Panel attrition: Separating stayers, fast attriters, gradual attriters, and lurkers. Sociological Methods & Research, 43(4):699–723

[41] Watson N Lynn P (2021) Refreshment Sampling for Longitudinal Surveys. In: Lynn P (Ed) Advances in Longitudinal Survey Methodology, P. 1–25

[42] Laurie H Lynn P (2008) The Use of Respondent Incentives on Longitudinal Surveys. Institute for Social and Economic Research, ISER Working Paper Series, No. 2008-42. University of Essex

[43] Toepoel V (2012) Effects of Incentives in Surveys. In: Gideon L (Ed) Handbook of Survey Methodology for the Social Sciences. Springer New York, New York, NY, P. 209–223

[44] Cabrera-Alvarez P Lynn P (2023) Increasing the value of an early bird incentive in a mixed-mode longitudinal survey. Understanding Society Working Paper Series 2023-11

[45] Grotophorst Z Bilgen I Dutwin D (2023) The Use of Data Cleaning Procedures in Probability-Based Panels. https://norc.org/content/dam/norc-org/pdfs/Data%20Clean-ing%20Procedures%20Research%20Brief.pdf (As at 11.03.2024)

[46] Public Health Evaluation and Impact Assessment Consortium (PHEIAC) (2013) Evaluation of the use and impact of the European Community Health Indicators ECHI by Member States. Final report. European Union

[47] Gabrys L Schmidt C Heidemann C(2018) Selecting and defining indicators for diabetes surveillance in Germany. J Health Monit 3(S3): 3–21. https://edoc.rki.de/handle/176904/5679 (As at 05.03.2024) 10.17886/RKI-GBE-2018-063 PMC8852787 35586543

[48] Heidemann C Paprott R Schmidt C(2019) Aufbau einer Diabetes-Surveillance in Deutschland – Ergebnisse der ersten Projektphase 2015 – 2019. Epid Bull 2019;45:473–478

[49] Thom J Walther L Eicher S(2023) Mental Health Surveillance am Robert Koch-Institut – Strategien zur Beobachtung der psychischen Gesundheit der Bevölkerung. Bundesgesundheitsbl 66(4):379–390 10.1007/s00103-023-03678-4 PMC9969389 36847853

[50] Zeiher J Varnaccia G Finger JD(2018) Einflussfaktoren der Adipositas im Kindesalter: das AdiMon-Indikatorensystem. Epid Bull 19:183–185

[51] Reitzle L Hansen S Paprott R(2018) National public health system responses to diabetes and other important noncommunicable diseases: Background, goals, and results of an international workshop at the Robert Koch Institute. Bundesgesundheitsbl 61(10):1300–1306 10.1007/s00103-018-2806-z 30191268

[52] Heidemann C Reitzle L Ziese T(2021) Diabetes-Surveillance am Robert Koch-Institut – Modellprojekt für den Aufbau einer NCD-Surveillance in Deutschland. Public Health Forum 29(4):277–281

