# Corrigendum: Health in Germany: Establishment of a population-based health panel

**DOI:** 10.25646/12077

**Published:** 2024-04-16

**Authors:** Johannes Lemcke, Julika Loss, Jennifer Allen, Ilter Öztürk, Marcel Hintze, Stefan Damerow, Tim Kuttig, Matthias Wetzstein, Claudia Hövener, Ulfert Hapke, Thomas Ziese, Christa Scheidt-Nave, Patrick Schmich

Lemcke J, Loss J, Allen J, Öztürk I, Hintze M et al. (2024)

Journal of Health Monitoring 9(S2): 2–21.

DOI 10.25646/11992

This article was initially published without an acknowledgement, this has now been added by the authors (see page 16).

The corrected version of the article is available at DOI 10.25646/11992.2

